# Supplementary figures and images for: Mosaic and cocktail capsid-virus-like particle vaccines for induction of antibodies against the EPCR-binding CIDRα1 domain of PfEMP1
Source: PLoS One. 2024 Jul 24;19(7):e0302243. doi: 10.1371/journal.pone.0302243 (PMC11268589; doi:10.1371/journal.pone.0302243)

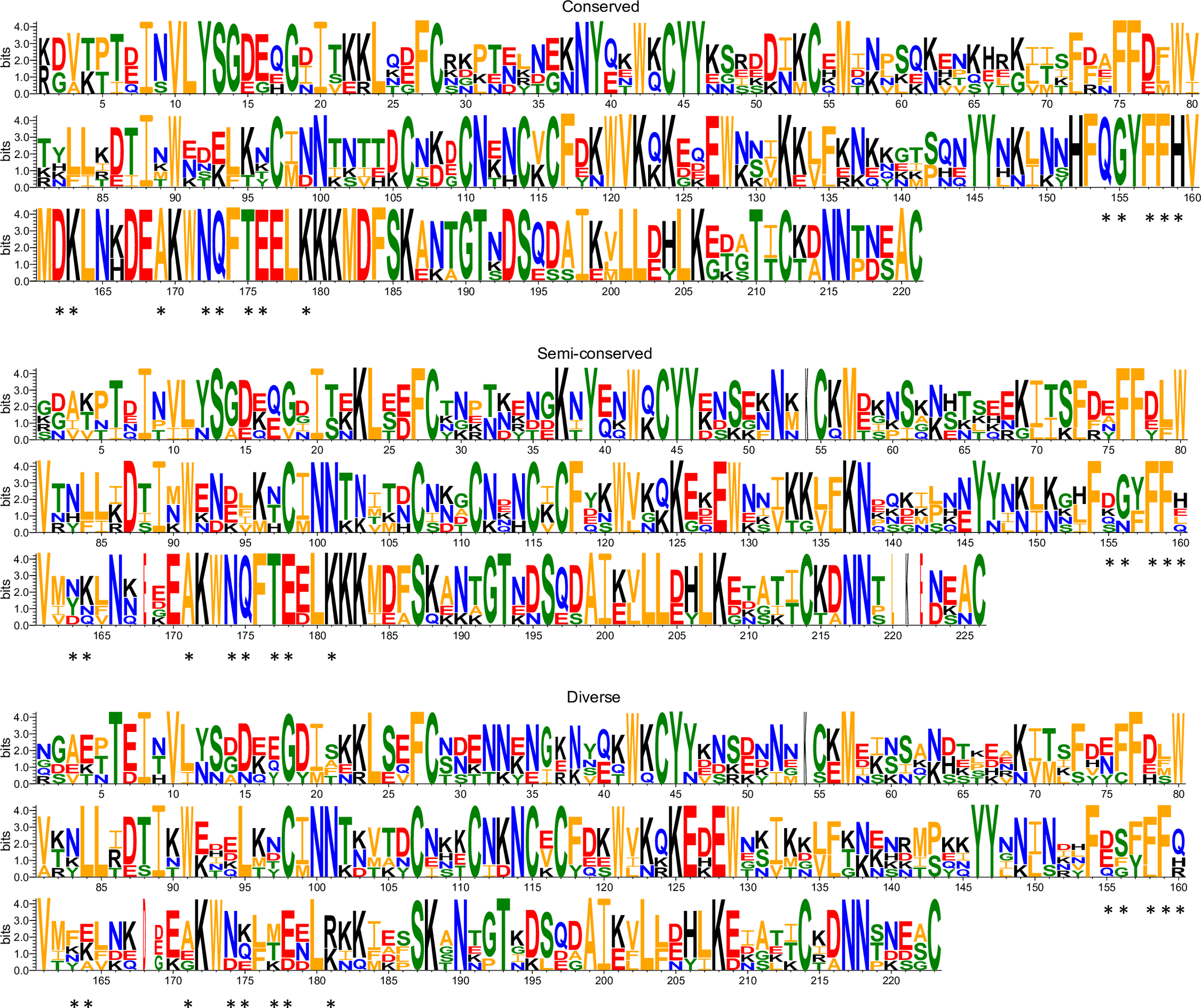

Supplement: S1 Fig — Logo were generated in WebLogo3 by alignment of the five CIDRα1 selected according to the respective vaccination strategy (conserved, semi-conserved and diverse). Asterisks indicate the 13 specified ammino acid positions constituting the putative epitope. (TIF) [file pone.0302243.s001.tif]

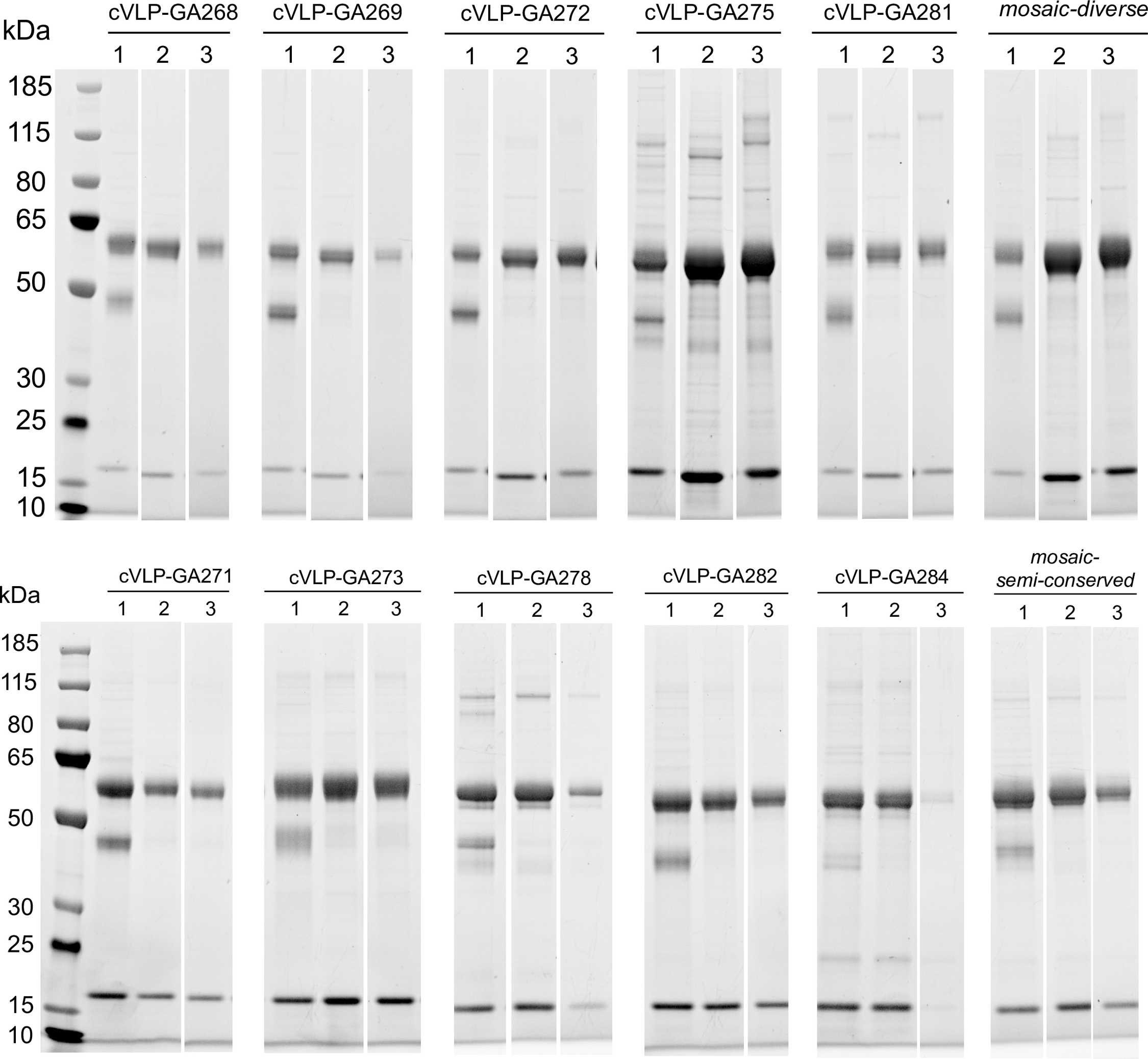

Supplement: S2 Fig — First lane (1) represents the samples pre-dialysis. The bands correspond to Tag-cVLP (16.5 kDa), unbound Catcher-CIDRα1 (~40 kDa) and coupled CIDRα1-cVLP (~56 kDa). The second lane (2) represents the samples post-dialysis and before spin test. The third lane (3) represents the samples post-dialysis and post-spin test. cVLP-GA275 and cVLP-GA284 were excluded from the study. The amount of cVLP-GA275 sample recovered after dialysis was insufficient to proceed with further analyses and immunization, while cVLP-GA284 demonstrated propensity to aggregation and instability upon performance of the spin test. Images are not cropped but stitched from multiple gel images. (TIF) [file pone.0302243.s002.tif]

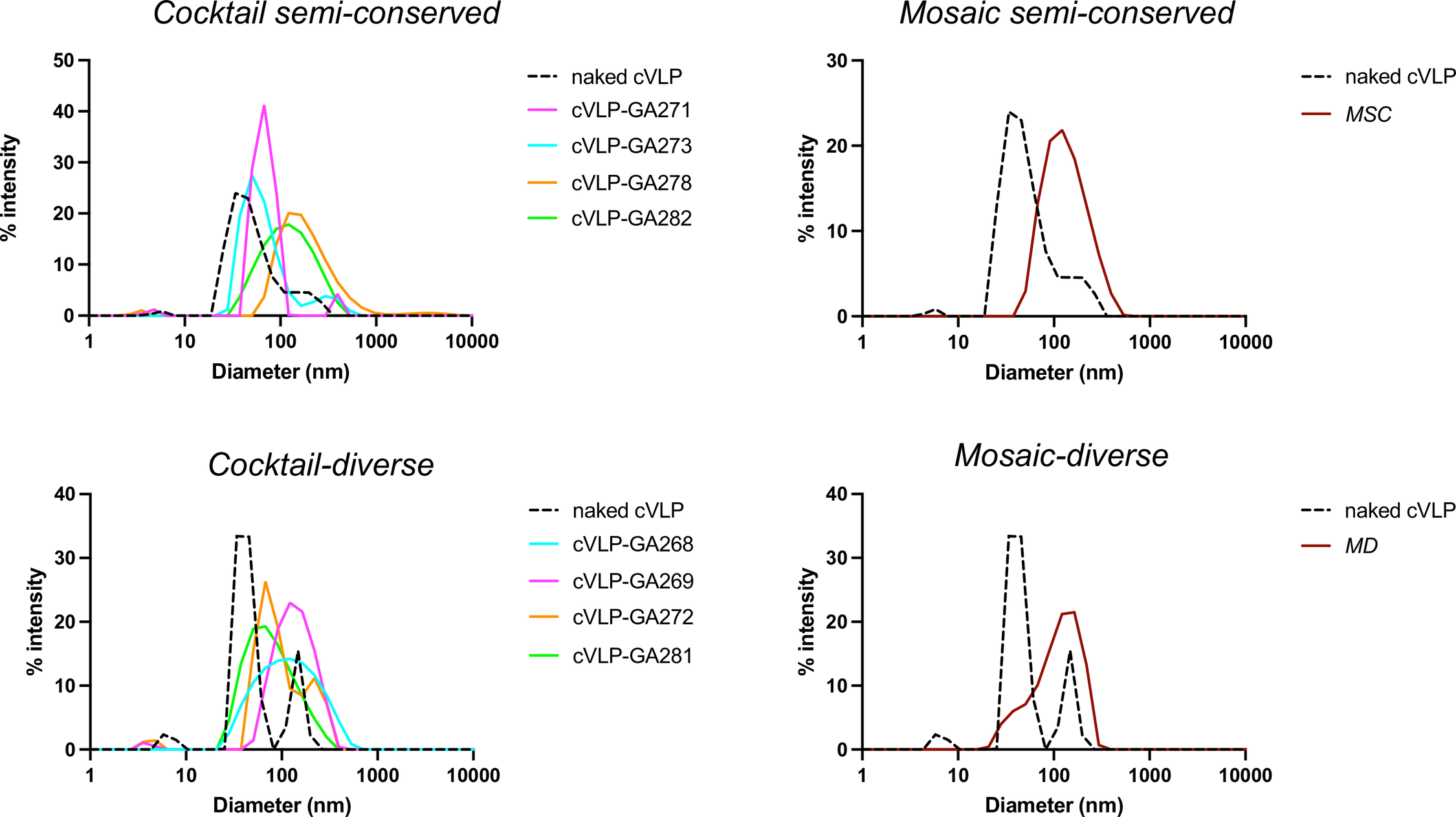

Supplement: S3 Fig — Naked SpyT-cVLPs (dashed line) predominant population shows 65.7 nm with 81.2% Pd in the cocktail- and -mosaic-semi-conserved-cVLPs and 41.9 nm with 20.5% Pd in the cocktail- and -mosaic-diverse-cVLPs. Cocktail-semi-conserved-cVLPs indicate a population size of 63.3–138.2 nm with 22.2–58.8% Pd, mosaic-semi-conserved-cVLPs show 146.8 nm and 53.1% Pd, cocktail-diverse-cVLPs 86.6–147.4 nm with 39.4–70.4% Pd and mosaic-diverse-cVLPs 120.3 nm with 49% Pd. Abbreviations: MD, mosaic-diverse; MSC, mosaic-semi-conserved. (TIF) [file pone.0302243.s003.tif]

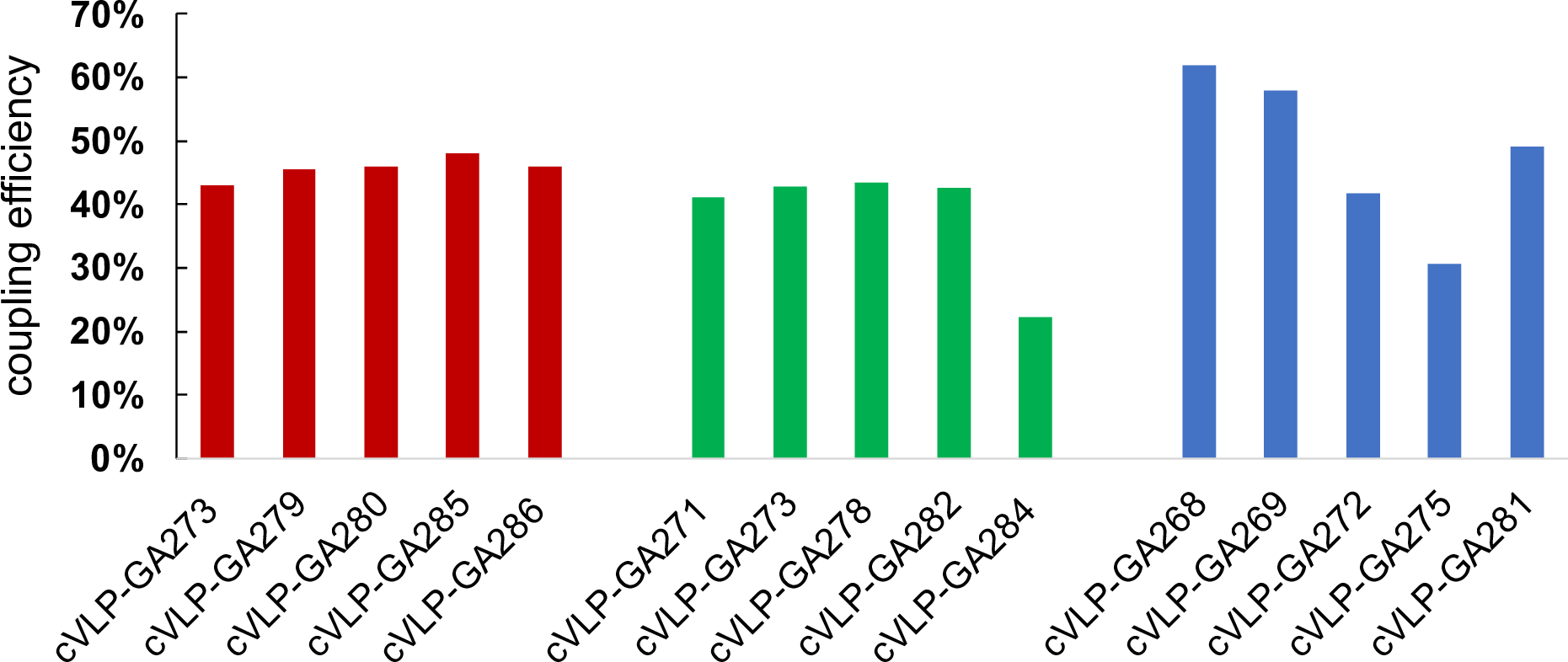

Supplement: S4 Fig — (TIF) [file pone.0302243.s004.tif]

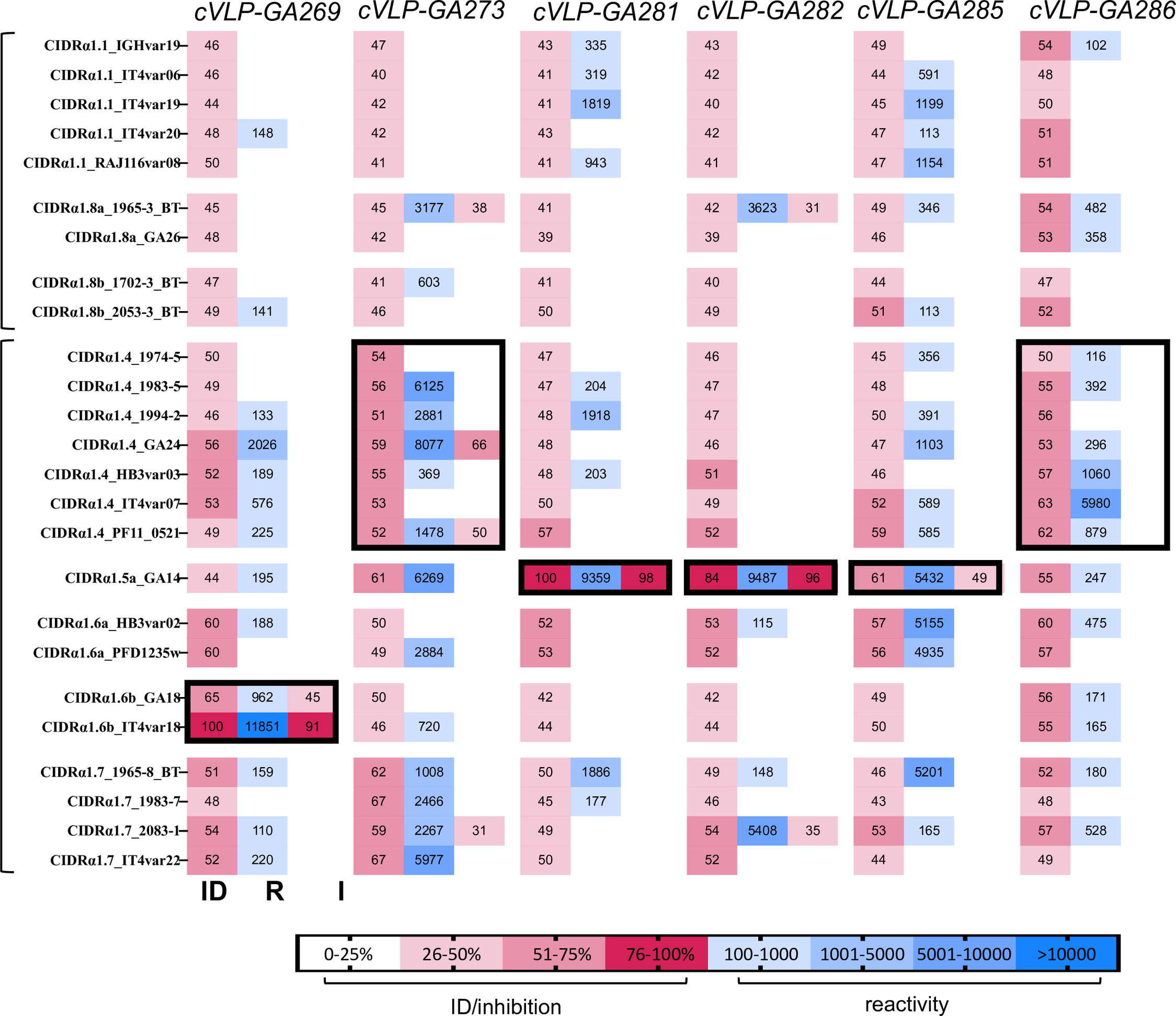

Supplement: S5 Fig — Heat map showing antibody responses to 25 CIDRα1 protein variants (rows). For each vaccination group (columns), percentage of identity (ID) between vaccine and test protein, IgG’s reactivity (R) towards the test protein variant measured as mean fluorescence intensity (MFI) and the IgG’s EPCR-binding inhibitory ability (I) are reported. Black rectangles identify CIDRα1 subgroups (CIDRα1.1–8) to which variants in the vaccine belong. (TIF) [file pone.0302243.s005.tif]

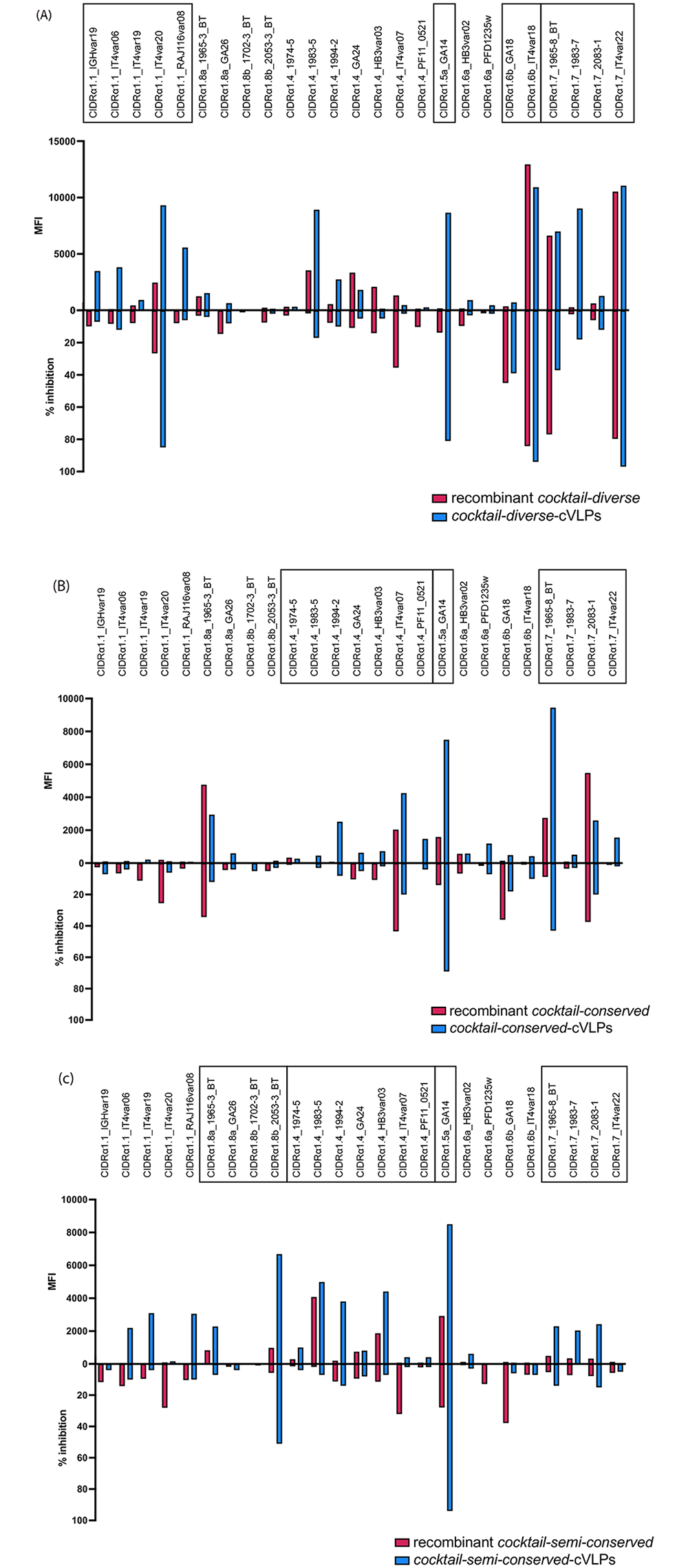

Supplement: S6 Fig — Antibody responses were tested against the same 25 CIDRα1 protein variants panel used to test immunogenicity of cVLP-based vaccines. Data analysis was carried out as before. Black rectangles identify CIDRα1 subgroups (CIDRα1.1–8) to which variants in the vaccine belong. Wilcoxon matched-pairs signed rank test resulted in significant reactivity differences in all three cases (recombinant vs cocktail-diverse-cVLPs: p-value = 0,0088; recombinant vs cocktail-semi-conserved-cVLPs: p-value <0,0001; recombinant vs cocktail-conserved-cVLPs: p-value = 0,0025). No significant differences in inhibition were found exploiting the same statistical test. (TIF) [file pone.0302243.s006.tif]

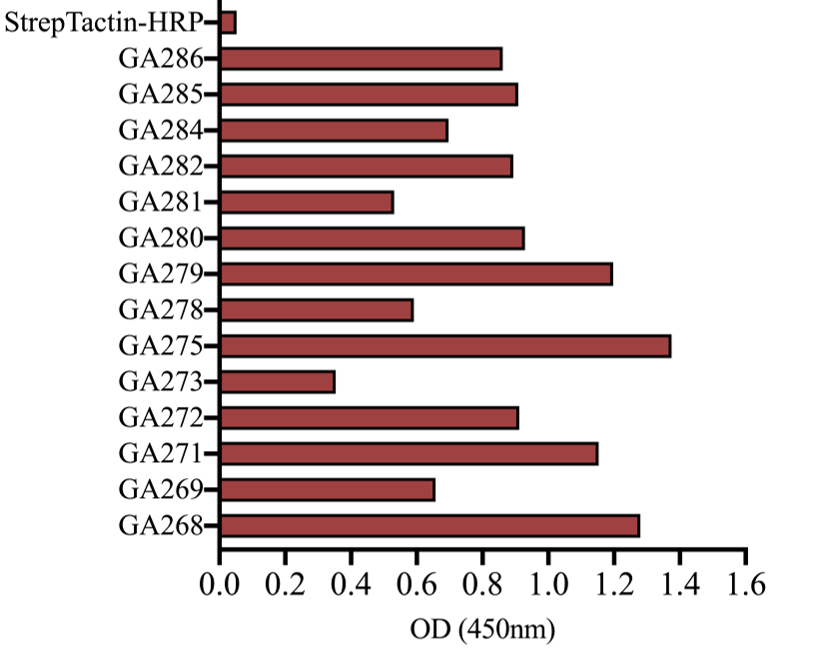

Supplement: S7 Fig — Plates were coated with recombinant EPCR at 3 μg/mL, recombinant proteins were added at 10 μg/mL, detection was performed with StrepTactin-HRP. OD was measured at 450 nm. Positive control: EPCR binding CIDRα1.4 domain, GA054. Negative control: StrepTactin-HRP only. (TIF) [file pone.0302243.s007.tif]
