# Supplementary material for: Mosaic and cocktail capsid-virus-like particle vaccines for induction of antibodies against the EPCR-binding CIDRα1 domain of PfEMP1
Source: PLoS One. 2024 Jul 24;19(7):e0302243. doi: 10.1371/journal.pone.0302243 (PMC11268589; doi:10.1371/journal.pone.0302243)
Supplement: S1 File — (DOCX) [file pone.0302243.s008.docx]

**Supplementary methods**

**Mass spectrometry**

The samples were dried in an Eppendorf Speedvac and re-solubilized in 20 μl of lysis buffer (6 M Guanidinium Hydrochloride, 10 mM TCEP, 40 mM Chloroacetamide, 50 mM HEPES pH 8.5). Samples were boiled at 95°C for 5 min, after which they were sonicated for 2.5 min. Samples were diluted to 60 μL with 10% Acetonitrile, 25 mM Tris pH 8.5. 100 ng LysC (MS grade, Wako) were added and samples were incubated at 37°C for 4 hours. Samples were further diluted to 200 μL with 10% Acetonitrile, 25 mM Tris pH 8.5, and 50 ng trypsin (MS grade, Promega) was added. Samples were incubated overnight at 37°C. Enzyme activity was quenched by adding 2% trifluoroacetic acid (TFA) to a final concentration of 1%. Prior to mass spectrometry analysis, the samples were acidified and desalted on SOLAμ^TM^ SPE plate (HRP, ThermoFisher). The eluted peptides were concentrated in an Eppendorf Speedvac, and re-constituted in 1% TFA, 2% Acetonitrile for Mass Spectrometry (MS) analysis**.**

For each sample, peptides were loaded onto a 2 cm C18 trap column (ThermoFisher 164705), connected inline to a 15 cm C18 reverse-phase analytical column (Thermo EasySpray ES803) using 100% Buffer A (0.1% Formic acid in water) at 750 bar, using the Thermo EasyLC 1200 HPLC system at 30 ^o^C. Peptides were eluted over a 70 minute gradient ranging from 6 to 60% of 80% acetonitrile, 0.1% formic acid at 250 nl/min, and the Q-Exactive instrument (Thermo Fisher Scientific) was run in the “DD-MS2 top10 method” setting. Full MS spectra were collected at a resolution of 70,000, with an Automatic Gain Control (AGC) target of 3Å~106 or a maximum injection time of 20 ms and a scan range of 300–1750 m/z. The MS2 spectra were obtained at a resolution of 17,500, with an AGC target value of 1Å~106 or maximum injection time of 60 ms, a normalised collision energy of 25 and an intensity threshold of 1.7e4. Dynamic exclusion was set to 60 s, and ions with a charge state <2 or unknown were excluded. MS performance was verified for consistency by running complex cell lysate quality control standards, and chromatography was monitored to check for reproducibility.

Raw data files were analysed using Proteome Discoverer 2.4. Label-free quantitation was enabled in the processing and consensus steps, and spectra were matched against a database of 245 common contaminants and the five proteins known to be present in the formulation. Dynamic modifications were set as Oxidation (M), and Acetyl on protein N-termini. Cysteine carbamidomethyl was set as a static modification. All results were filtered to a 1% FDR, and protein quantitation was done using the built-in Minora Feature Detector. From the list of returned peptides we only considered abundances of peptides matching to one protein uniquely. Percentage of each coupled CIDRα1 was computed as the ratio of unique peptides abundance and total peptides abundance.

**IgG reactivity and EPCR-binding inhibition**

Reactivity raw values were subtracted a cutoff established as two standard deviations from the mean MFI signal of pooled IgGs, from 20 randomly selected mice sera collected 13 days prior vaccination. All negative values resulting from this operation were set to zero to simplify visualization of plots. Inhibition of EPCR binding toward each protein array variant was expressed in percentage, by comparing the signal from biotinylated EPCR to each variant without or in the presence of mouse plasma IgG, as previously described (1).

1. Harmsen C, Turner L, Thrane S, Sander AF, Theander TG, Lavstsen T. Immunization with virus-like particles conjugated to CIDRα1 domain of Plasmodium falciparum erythrocyte membrane protein 1 induces inhibitory antibodies. Malar J. 2020;19(1):1–11.
